# Nonlinear shear characteristics of frozen loess-concrete interface

**DOI:** 10.1371/journal.pone.0290025

**Published:** 2023-08-15

**Authors:** Qingwei Zhang, Chunguang Zhang

**Affiliations:** 1 School of Civil Engineering and Architecture, Anyang Normal University, Anyang, Henan, China; 2 CTG Operation and Administration Center for River Basin Hydro Complex, China Three Gorges Corporation, Yichang, Hubei, China; University of Birmingham, UNITED KINGDOM

## Abstract

Under the different temperature environment, the precast pile-soil interface characteristics has an important impact on the safety and long-term stability for pile foundation. A large precast pile-soil shear experimental device is used to carry out the direct shear test of concrete-loess interface with different moisture contents under different freezing temperatures. The variation laws of shear strength parameters are revealed with influencing factors, and the shear mechanism of interface is discussed. The stress-strain constitutive equation of interface is proposed, and the shear strength criterion is established with considering the effects of temperature and moisture content on cohesion and internal friction angle. The results show the curve of shear stress and shear displacement can be divided into three stages: elastic deformation stage, plastic deformation stage and sliding failure stage, which macroscopically reflects the shear failure mechanism of the frozen soil-concrete interface. The shear strength of the interface is affected by the test temperature, sample moisture content and normal stress. The lower the test temperature, the greater the shear strength of the interface; With the increase of normal stress, the shear strength of interface increases; With the increase of moisture content, the shear strength of the interface increases and then decreases. The relationship of shear stress and shear displacement of frozen soil-concrete interface can be well described by the piecewise combination of hyperbolic function and linear function.

## 1. Introduction

Pile foundation has been widely used in highway, railway, bridge and high-rise building projects, and its function is to transfer the superstructure load to the rock or soil layer with high strength and low compressibility through the pile body, which provides higher bearing capacity and reduces foundation settlement. The stress and deformation between soil and structure are mainly transmitted through their interface [1]. Because the interface is affected by structure and soil, it shows more complex mechanical properties, which are different from both soil properties and structural material properties. And the unique mechanical characteristics of the interface will have an important impact on the stress, deformation and interaction of the structure and soil, even the weak link and the key area of the stability of the structural system. Therefore, the interface between soil and structure should be considered specially as the interaction between soil and structure is analyzed [2–4]. Especially in cold regions, the shear characteristics of frozen soil-structure interface are important basis for analyzing the performance of engineering structures.

The model pile test can effectively simulate the action of the different types of pile-soil under different loads, and can evaluate the limit states of failure and deformation [5, 6]. Because of the wide applicability and relatively simple test device, the direct shear method is widely used to study the mechanical properties of precast pile-soil interface. The strength of the soil-structure interface mainly refers to the shear strength, which is mainly affected by the type of soil [7, 8], roughness of structure surface [9–11], ambient temperature [12], etc. Among them, because of the regional differences and seasonal changes, it is particularly important for the contact strength of soil-structure interface affected by the ambient temperature.

Under the influence of ground temperature, Yavari et al. [13] and Li et al. [14] tested the friction behavior of pile-clay interface through direct shear test. The results show that with the temperature increasing, the internal friction angle of pile-clay interface increases and the cohesion decreases. However, Donna et al. [15] obtained the contrary results, the results show that the internal friction angle of pile-clay interface decreases and the cohesion increases with the increase of temperature. Saeed et al. [16] conducted a series of direct shear tests with a temperature controlled direct shear tester to analyze the effect of thermal cycle on pile-soil interface strength.

In the freezing environment, Zhao et al. [17] adopted large multifunctional direct shear instrument to measure the cyclic-direct shear characteristics of artificial frozen soil-structure interface under four kinds of normal stress and four kinds of negative temperature. Shi et al. [18] adopted direct shear instrument to carry out a series of tests to explore the mechanism of freezing strength of the interface between frozen fine sand and structure. Shi et al. [19] conducted low temperature direct shear tests with different initial water content (14%, 16%, 18%) and initial void ratio (1, 0.8 and 0.6) at different temperatures (-1°C, -2°C and -3°C). Liu et al. [20] carried out a series of direct shear tests by a large temperature controlled direct shear test system (TZJ-150), and the mechanical properties of frozen soil-concrete interface are analyzed. The curve of shear stress and shear displacement is divided into five parts: elastic deformation, plastic deformation, overall sliding, strain hardening and stable residual strength. Wang et al. [21] conducted an experimental study on the shear stress response and shear strength index of soil-structure interface under frozen soil conditions by using the improved roughness algorithm. The effects of roughness, temperature, moisture content and vertical stress on the evolution of mechanical properties are deeply analyzed. He et al. [22, 23] analyzed the effect of freeze-thaw cycle on the shear properties of frozen soil-concrete interface with direct shear test. The influences of test samples are composed of initial moisture content of 9.2%-20.8%, temperatures (-1°C, -3°C, -5°C) and freeze-thaw (F-T) cycles (0, 5, 10, and 20).

Although many scholars have done a lot of research on the frozen soil-structure interface, there is no in-depth and systematic research on the influence factors of its shear properties. Therefore, in this paper, the mechanical relationship of frozen soil-concrete interface is simplified as a two-dimensional contact surface between frozen soil and structure. The mechanical properties of frozen soil-concrete interface are analyzed through the direct shear tests, which are carried out under different test temperatures, sample moisture content and normal stress. The shear stress mechanism of interface is discussed, the stress-strain constitutive equation of interface is proposed, and the shear strength criterion is established with considering the effects of temperature and moisture content.

## 2. Test system

### 2.1 Test principle

The stress and strain of the precast pile-soil interface is observed during the pile-soil interface shear simulation test, the generation and development mechanism of the shear characteristics are analyzed to discuss the stability of the pile foundation under the high and low temperature environment.

At the beginning of the shear test, the pile-soil test box is placed on the pile simulation box, the soil is filled and compacted, and a loading plate is covered, then a vertical load is applied on loading plate to simulate the earth pressure. The horizontal thrust is applied to the pile simulation box to make the pile simulation box slide between the operation platform and the precast pile-soil test box, and the precast pile-soil interface produces relative displacement and shear failure until the horizontal thrust tends to be stable. During the test, the displacement and horizontal thrust of the pile simulation box are measured and recorded.

Because the concrete slab is located at the lower part of compacted soil, while the horizontal thrust acts on the concrete slab along the interface direction, the concrete slab is also subjected to the vertical pressure transmitted by the vertical load through the compacted soil. The mechanical response of precast pile-soil interface occurs along the horizontal thrust direction. When shear displacement occurs, the pile-soil contact interface generates friction to balance the horizontal thrust. Assuming that the friction at the interface is evenly distributed, the interface friction corresponding to the maximum horizontal thrust is the ultimate friction (shear strength) at the precast pile-soil interface.

### 2.2 Test device

**Fig 1:**
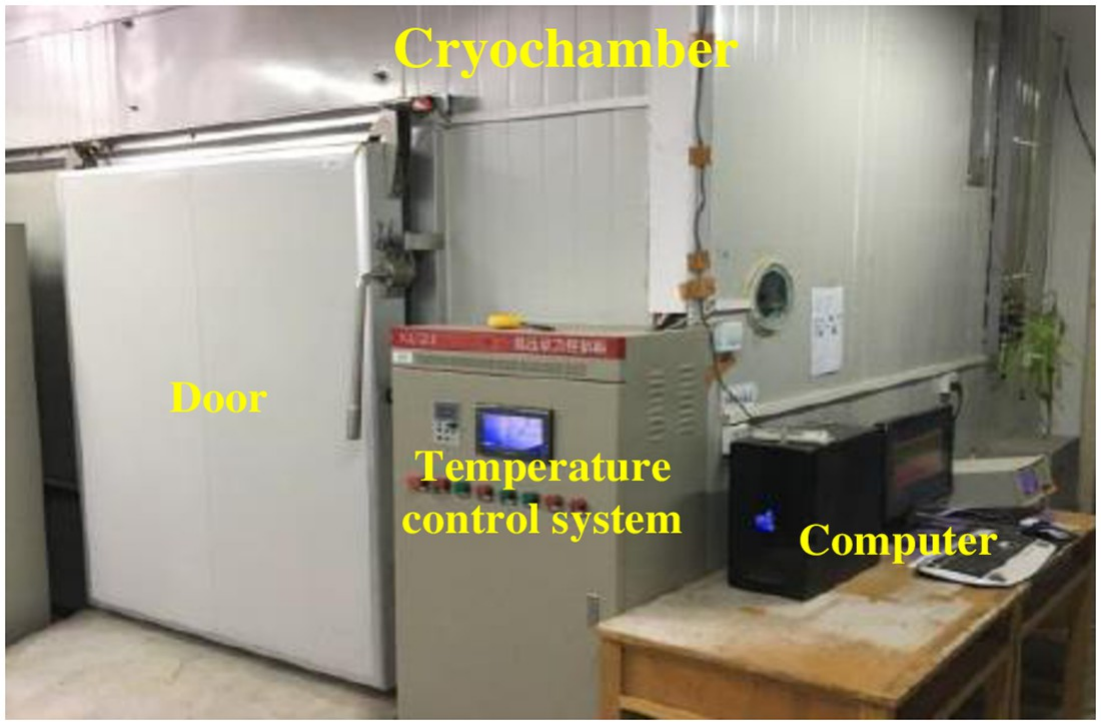
Low temperature laboratory.

Low temperature laboratory (as shown in Fig 1): the minimum temperature can reach -20°C, the indoor temperature can be automatically controlled, and the temperature control accuracy is ±0.5°C. The ambient temperature is measured in real time during the test process to make it as close to the set temperature as possible.

**Fig 2:**
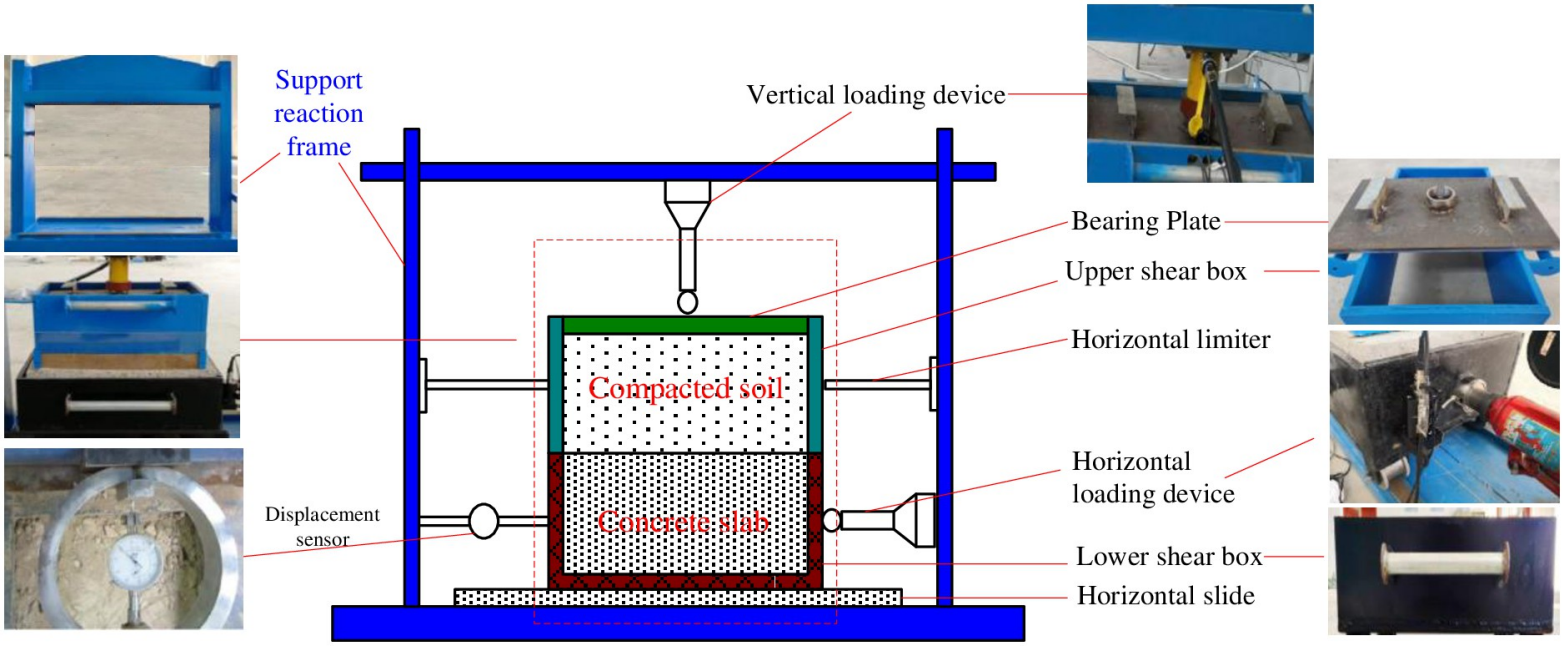
Large-scale pile-soil shear experimental device.

The large-scale precast pile-soil shear experimental device (as shown in Fig 2) is mainly composed of support reaction frame, shear box, horizontal loading system, vertical loading system, measurement system and data acquisition system. The test operation platform is composed of 130mm thick concrete slab and 20mm thick guide rail steel plate, which are clamped with bolts, and the plane size is 680×600mm. There are two V-shaped sliding grooves on the guide rail steel plate, and balls are evenly placed in the sliding groove to reduce friction. (1) The support reaction frame is made of high-strength steel, which has the advantages of large load, small deformation and high stability. (2) The pile simulation box is made of rectangular steel box and cast-in-situ C40 plain concrete, and the effective surface area of concrete slab is 610×600mm, which can bear the load of 10t and ensure it stability and no-deformation under ultimate load. There is a V-shaped sliding groove at the bottom of the steel box (corresponding to the guide rail steel plate). (3) The external dimension of pile-soil test chamber is 680×600×300mm, the internal size is 610×600×300mm. The upper and lower openings of the box are made of 15mm thick steel plate, the edge is strengthened, there is a support frame on the left, and a loading plate of the same material is attached. (4) The horizontal loading device is composed of gear mechanical jack, strain sensor and reaction frame, which can provide any load in the range of 0~100kN. The jack is equipped with a T-shaped handle of appropriate size, and the load can be applied evenly by rotating the handle. The measuring range of gear mechanical jack is 10t, and the strain sensor is a force measuring ring with class 1 accuracy of 100kN. (5) The vertical loading device is composed of hydraulic separated jack, strain sensor and reaction frame, which can be flexibly controlled to provide any load in the range of 0~100kN. The range of hydraulic separated jack is 10t, and the strain sensor is a force measuring ring with grade 1 accuracy of 100kN. (6) The displacement sensor consists of a dial indicator with a minimum measuring range of 0.01mm and a magnetic support.

## 3. Test setup

### 3.1 Test soil sample

**Fig 3:**
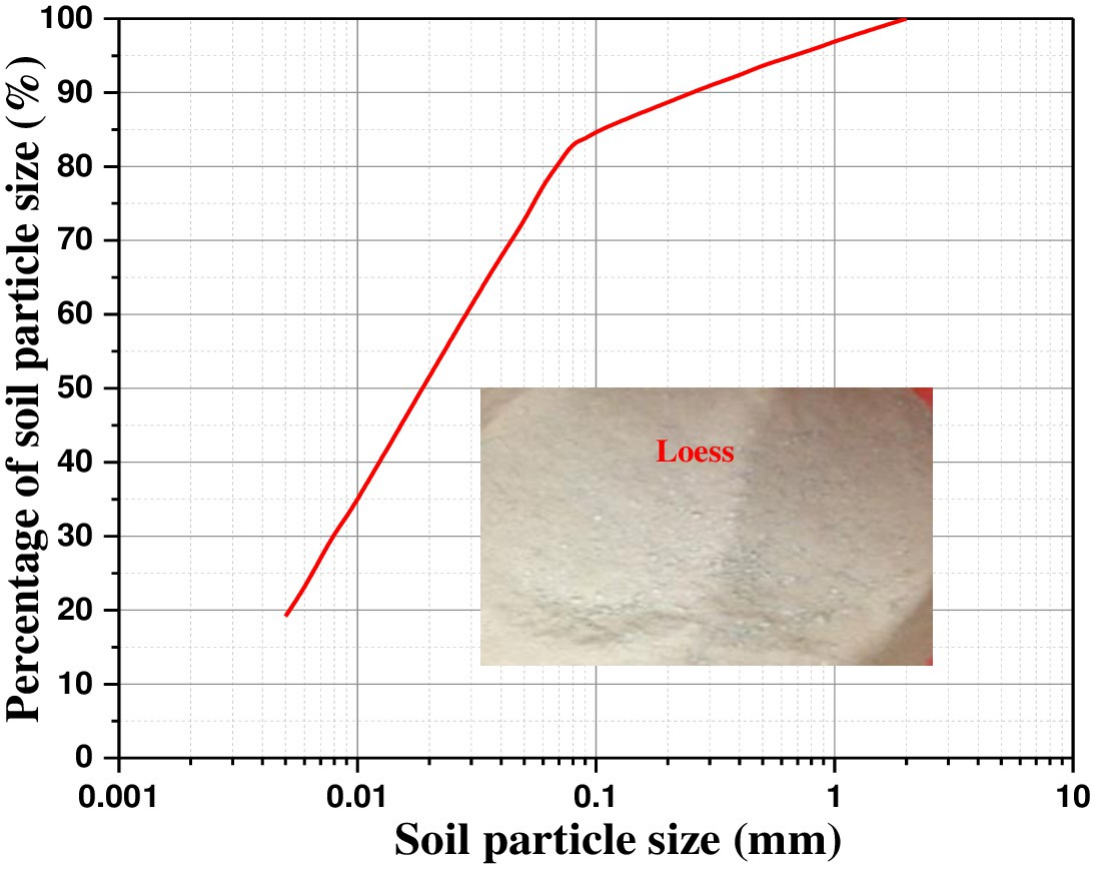
Particle size grading curve.

Test soil sample of loess is taken from Xining, Qinghai Province. All soil shall pass through 2mm sieve, and the soil sample used in the test shall be taken off the sieve (Fig 3). According to the standard for soil test method (GB/T50123-1999), the basic physical properties of soil samples are tested. According to the compaction test, liquid-plastic limit test and particle gradation test, the optimal moisture content of the test soil is 17.8%, the dry density is 1.89g/cm^3^, the liquid limit is 27.1%, the plastic limit is 12.2%, and the plasticity index is 10.6. The test soil belongs to silt. The cumulative curve of particle size grading of soil sample is shown in Fig 3.

### 3.2 Preparation of concrete sample

**Fig 4:**
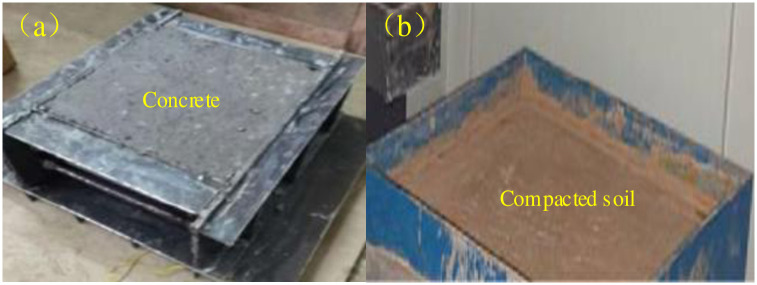
Concrete-soil sample preparation.

The concrete plate for the test is high-strength plain concrete, which is made with the same volume as the lower shear box (Fig 4a). The concrete is made of ordinary portland cement, sand, gravel, water and admixtures according to the mix ratio of 477:650:1107:176:5.72. The pouring mold is a self-developed removable steel mold, as shown in Fig 4a. The concrete sample shall be demoulded after curing in the standard curing room for 2 days, and continue to be cured at room temperature for 28 days.

### 3.3 Sample preparation

The soil sample is placed in the oven for more than 12 hours to dry the water in the soil. Then the dried soil sample is taken out to place it in a sealed container for cooling. And the amount of soil and water are calculated according to the volume of the shear box and the standard of 95% compactness. A certain amount of soil and water is taken for full mixing, which is placed in a sealed container for 12 hours to make the water fully and evenly.

During concrete-soil sample preparation, the concrete plate shall be placed at the bottom of the sample preparation box, and then the evenly mixed soil sample shall be paved above the concrete in layers and be compacted (Fig 4b). The outer part of the sample box with samples shall be covered with plastic bags and placed in the low temperature laboratory. After freezing for 24h at the set freezing temperature, the shear test shall be started (In the test preparation stage, a thermometer was buried at the interface of soil and concrete to observe the temperature change. It was found that the temperature at the interface of soil and concrete was basically stable after about 20 hours at the set temperature in the low-temperature laboratory.).

## 4. Concrete-soil shear stress test results and analysis

For the influence factors of shear mechanical properties of frozen soil-concrete interface, a series of shear tests are carried out with loading rate 1.0mm/min under five kinds of temperature, four kinds of normal pressure and four kinds of moisture content. The temperatures are set as 25°C, -2°C, -5°C, -8°C, -12°C, the normal stresses are set as 50kPa, 100kPa, 150kPa and 200kPa, and the water contents of the configured soil sample are set as 14%, 18%, 22% and 26%.

### 4.1 Shear stress and shear displacement test curve

**Fig 5:**
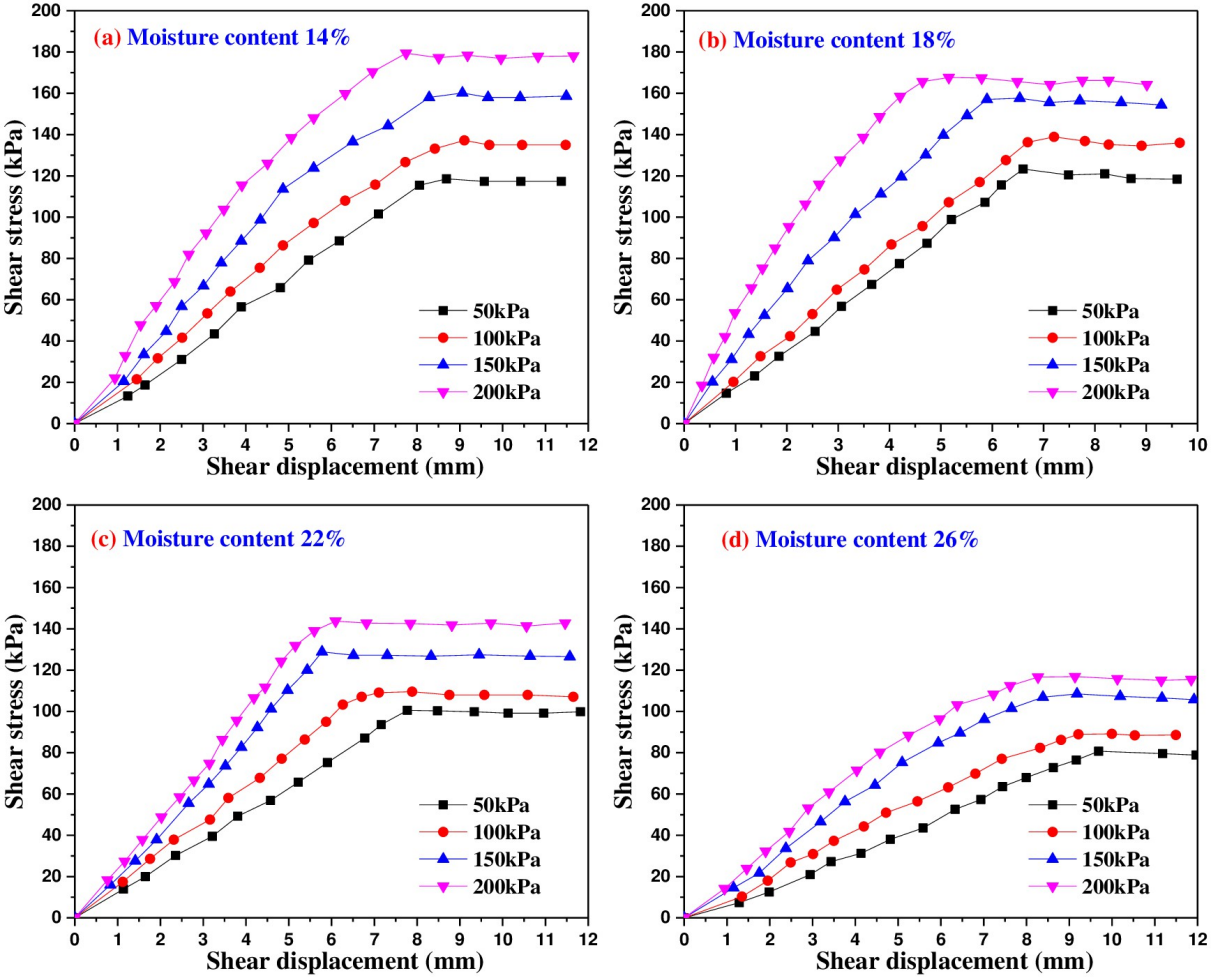
Shear stress and shear displacement curves of soil-concrete interface at 25°C.

**Fig 6:**
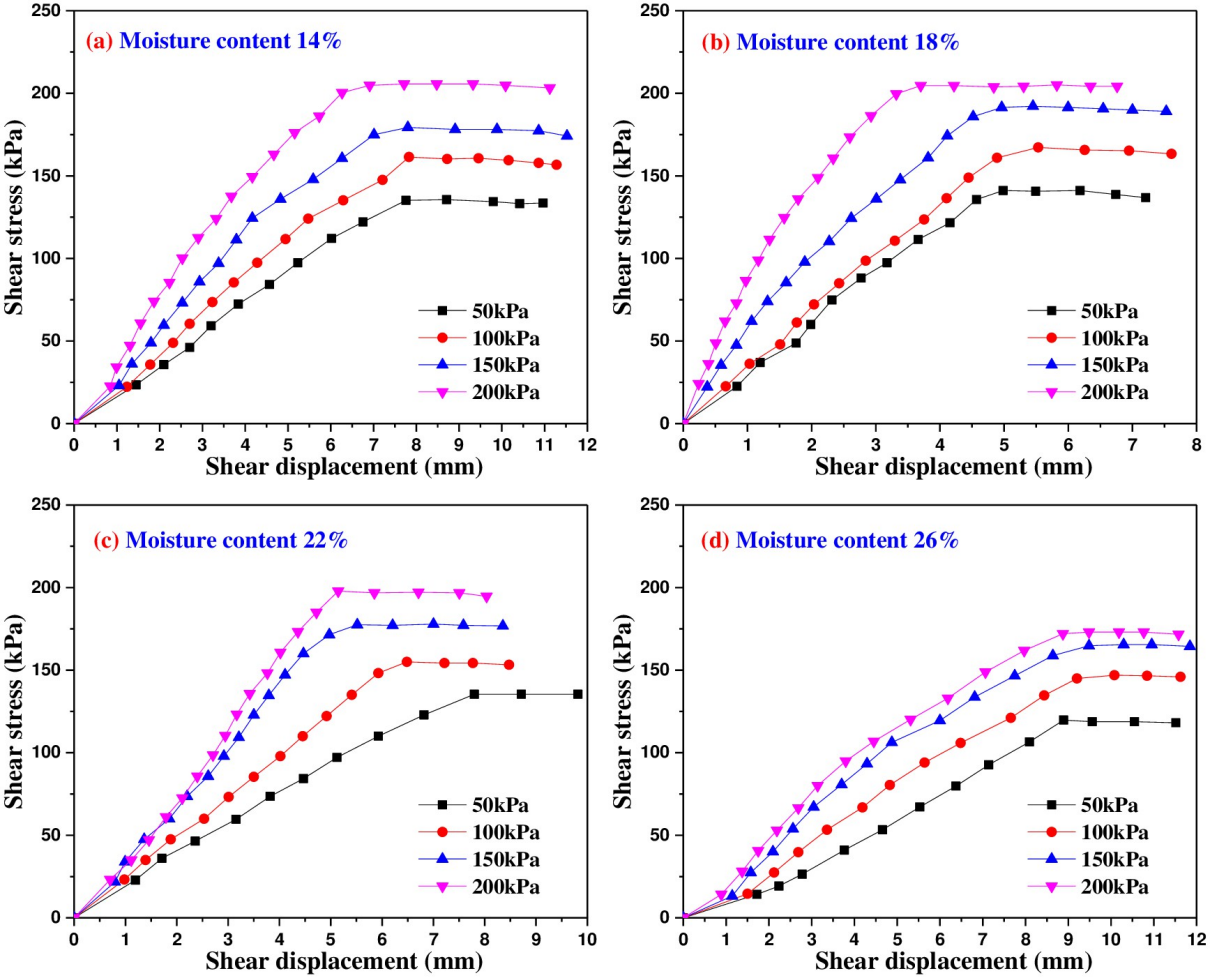
Shear stress and shear displacement curves of soil-concrete interface at -2°C.

**Fig 7:**
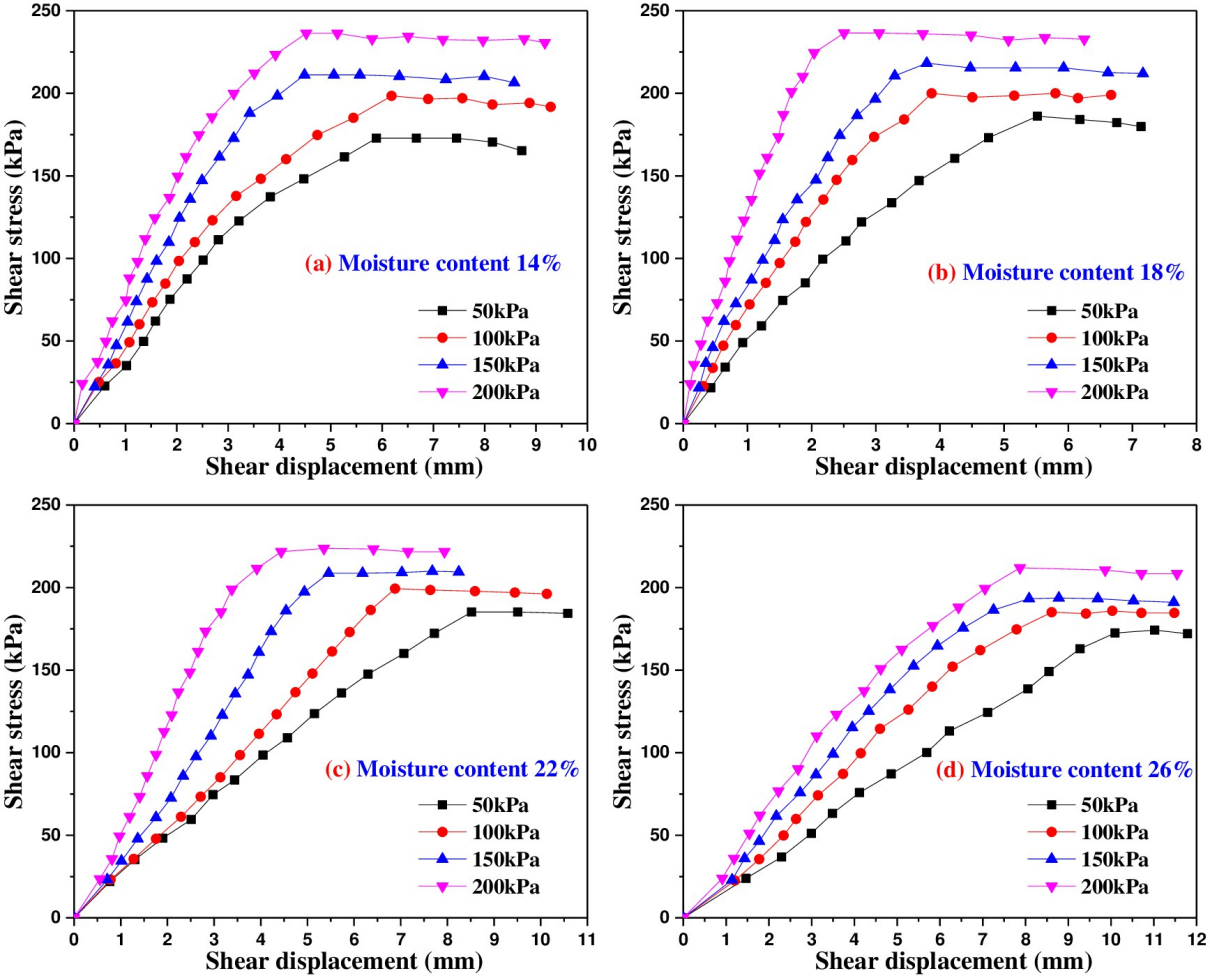
Shear stress and shear displacement curves of soil-concrete interface at -5°C.

**Fig 8:**
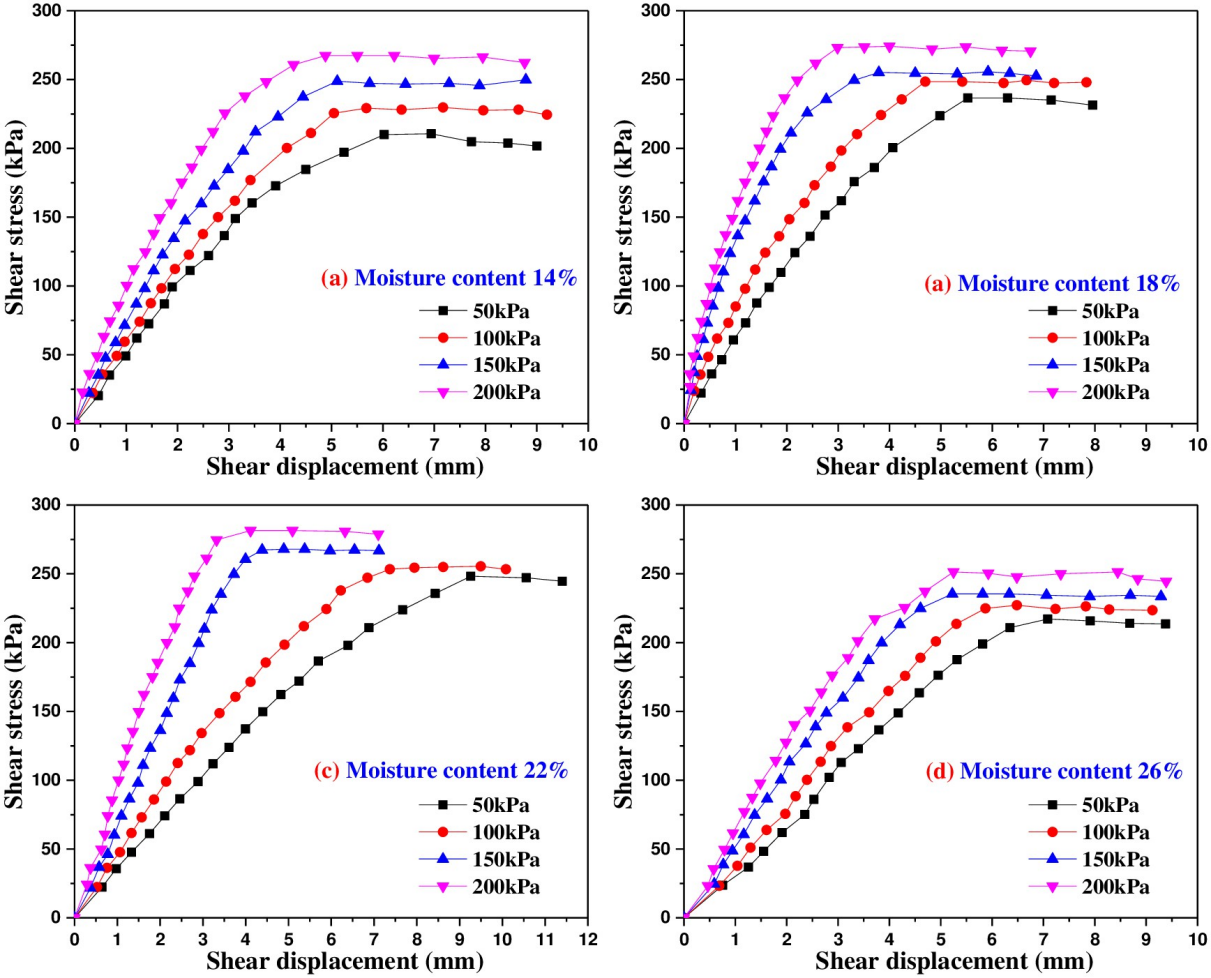
Shear stress and shear displacement curves of soil-concrete interface at -8°C.

**Fig 9:**
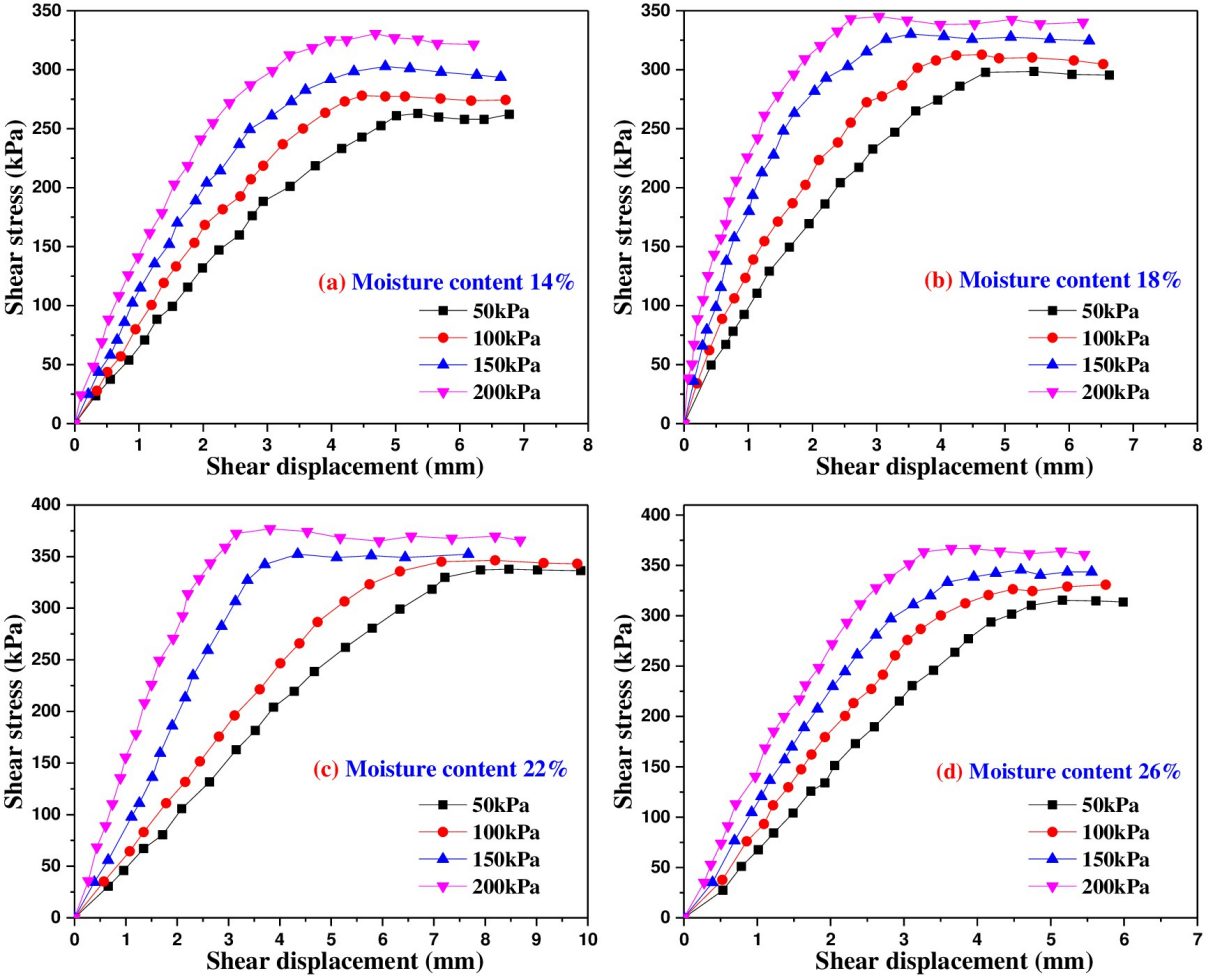
Shear stress and shear displacement curves of soil-concrete interface at -12°C.

Figs 5–9 are the variation curves of horizontal shear displacement and shear stress in direct shear test of frozen soil-concrete interface. According to the shape characteristics of the curves, it can be divided into three stages: elastic deformation stage, plastic deformation dominant stage and sliding failure stage. Under the action of small load, the contact surface is mainly elastic deformation, and the relationship of shear stress and displacement is close to a straight line. With the increase of shear stress, the plastic deformation gradually develops, and the relationship of shear stress and displacement presents a curve development. When the shear stress increases to a certain value, the shear displacement increases sharply, which means that the overall relative movement of the interface occurs, the stress is almost constant and the strain increases gradually.

### 4.2 Test phenomena

**Fig 10:**
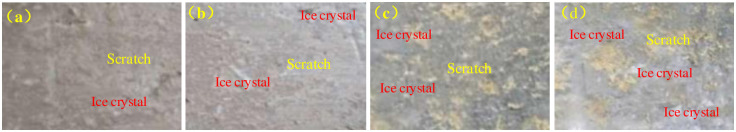
Concrete shear plane after direct shear (a) -5°C, 150kPa and 14% (b) -5°C, 150kPa and 18% (c) -5°C, 150kPa and 22% (d) -5°C, 150kPa and 26%.

The characteristics of concrete surface after shearing are shown in Fig 10. After shearing, scratches along the shear direction will be formed on the frozen soil surface. It can be seen that there are different degrees of residual soil samples on the concrete surface after shearing. Among them, there are more residual soil samples on the concrete surface after shearing under the condition of higher moisture content. In Fig 10, the area where the soil sample turns white represents the formation of ice crystals after water freezing. Scratches are more obvious under low water content conditions, that is, strip marks appear along the sliding direction, while blocky scratches appear at high water content. At the same time, more large scratches present on the soil interface with lower moisture content (14%, 18%, Fig 10a and 10b), however, less scratches present on the soil interface with higher moisture content (22%, 26%, Fig 10c and 10d). This phenomenon is related to the stress on the interface in the shear process. The shear strength of the interface is composed of cohesion and friction, and the cohesion of the interface is mainly borne by the cementation of ice crystals. When the moisture content is larger, the frozen interface will produce a significant cementation force, and ice crystals and unfrozen water will more fill the pores of the interface which strengthens the cementation and increases the particle friction of the interface, and then the friction of the interface increases. Therefore, when the moisture content of the soil sample is higher, there will be less scratches on the surface of the soil sample after shearing, and more soil particles will remain on the concrete surface. On the contrary, when the water content is lower, the amount of ice cementation is smaller, which results in less residual soil particles.

### 4.3 Shear strength of interface

The shear strength of the interface is a strength index as the shear and compressive strength of frozen soil, it is a function of soil sample dry density, external load action, moisture content, temperature and particle composition.

**Fig 11:**
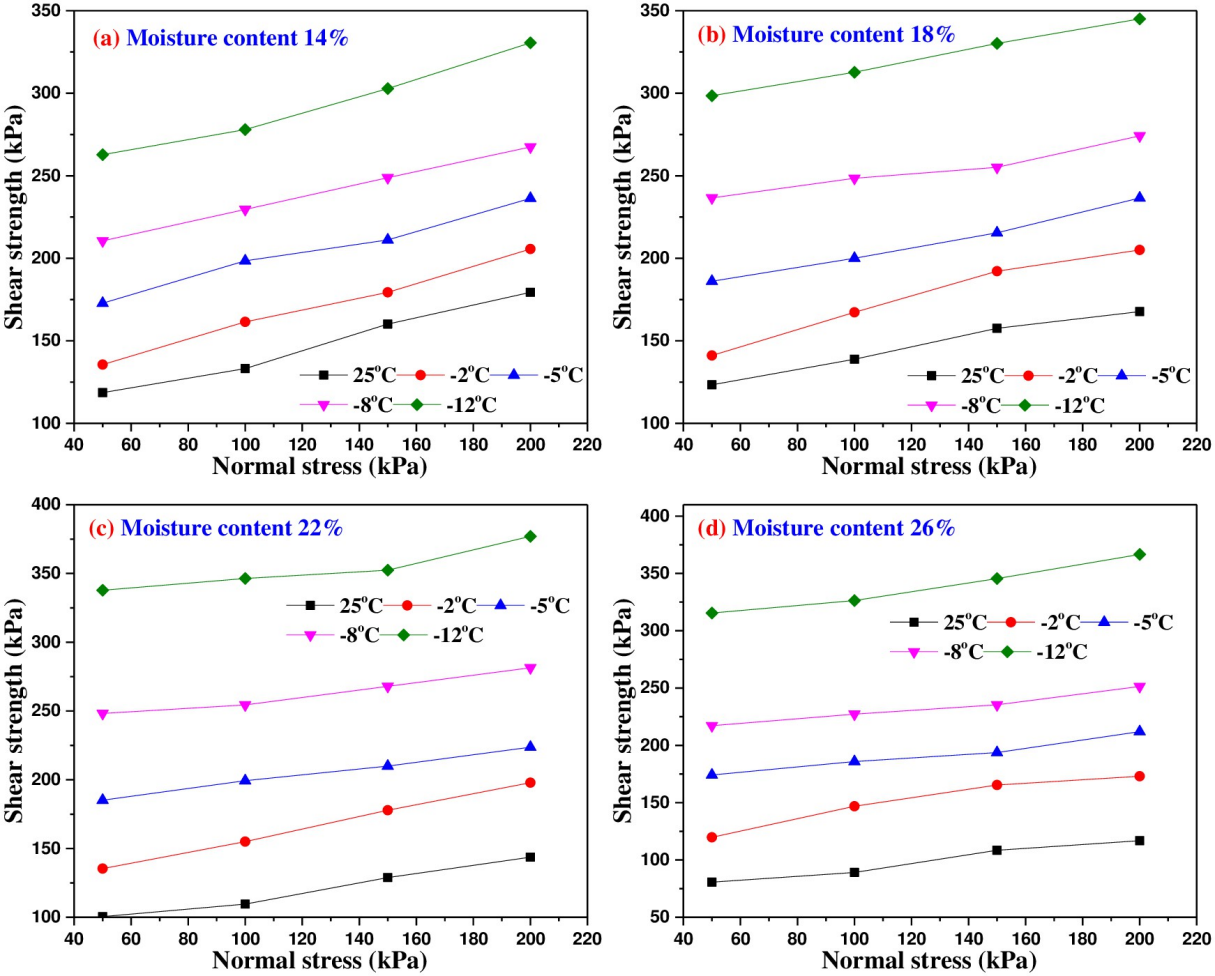
Variation curves of shear strength of the interface with different normal stress.

Fig 11 show the relationship curve between the shear strength and normal stress of frozen soil-concrete interface under different temperatures. It can be seen from Fig 11 that with the decrease of freezing temperature, the shear strength of the interface increases significantly. Moreover, with the moisture content of frozen soil increasing, the influence of freezing temperature on the shear strength is more obvious. The characteristics of soil-pile interface with temperature decreasing are beneficial to the safety and long-term stability of pile foundation. At the same moisture content, the lower the freezing temperature, the bigger the shear strength. This is mainly related to the influence of temperature on the shear strength of the interface, and the freezing temperature will directly affect the amount of unfrozen water content. When the freezing temperature decreases, the unfrozen water content near the interface decreases, the amount of ice crystals and cementation strength increase. When the moisture content increases from 22% to 26%, the effect of freezing temperature on shear strength increases with the moisture content increasing, that is, the freezing strength increases with the decrease of freezing temperature. It is calculated that the saturated moisture content of the loess sample in this test is 24.4%, so the soil sample with moisture content of 26% has reached saturation. Under the freezing temperature, the pores are completely filled with unfrozen water and ice crystals, and the ice cementation capacity reaches the maximum. The further increase of moisture content cannot continue to increase the ice cementation capacity. On the contrary, as the temperature decreases, ice crystals and frozen water fill the pores of the interface, which will reduce the friction of the interface.

It can also be seen from Fig 11 that under the same freezing temperature, the shear strength of frozen soil-concrete interface increases approximately linearly with the increase of normal stress. When the normal stress is smaller, the shear strength of frozen soil-concrete interface changes significantly with the increase of soil moisture content. With the increase of normal stress, the influence of moisture content on shear strength decreases. This is because under the bigger normal stress, the friction reduction effect enhances with the increase of moisture content.

**Fig 12:**
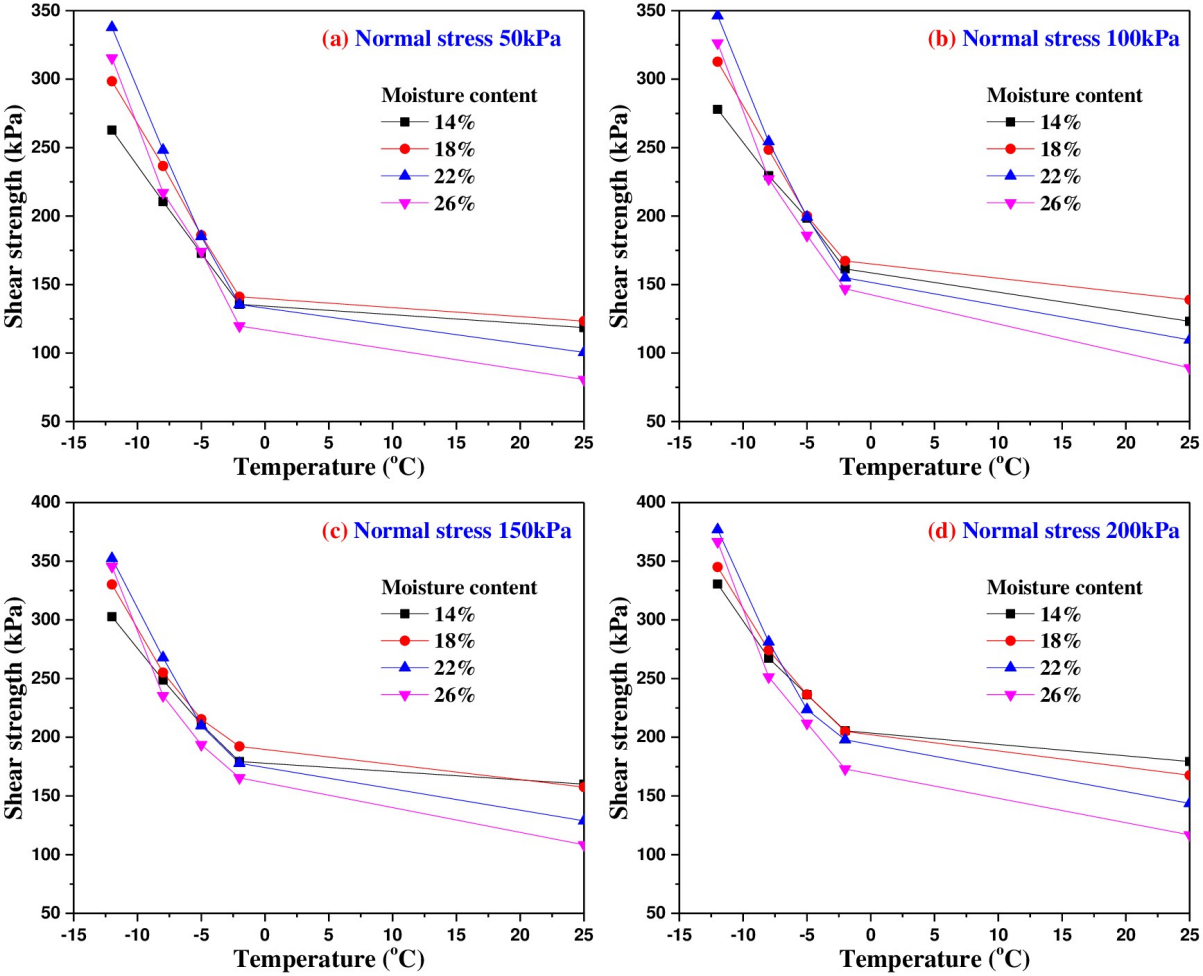
The relationship curves of shear strength and freezing temperature under different normal stresses.

Fig 12 shows the relationship between shear strength and freezing temperature under the same normal stress. It can be seen from Fig 12 that the shapes of shear stress and freezing temperature curves are very similar with different moisture content. With the decrease of freezing temperature, the shear strength of interface increases gradually, and the relationship is approximately linear under freezing temperature.

**Fig 13:**
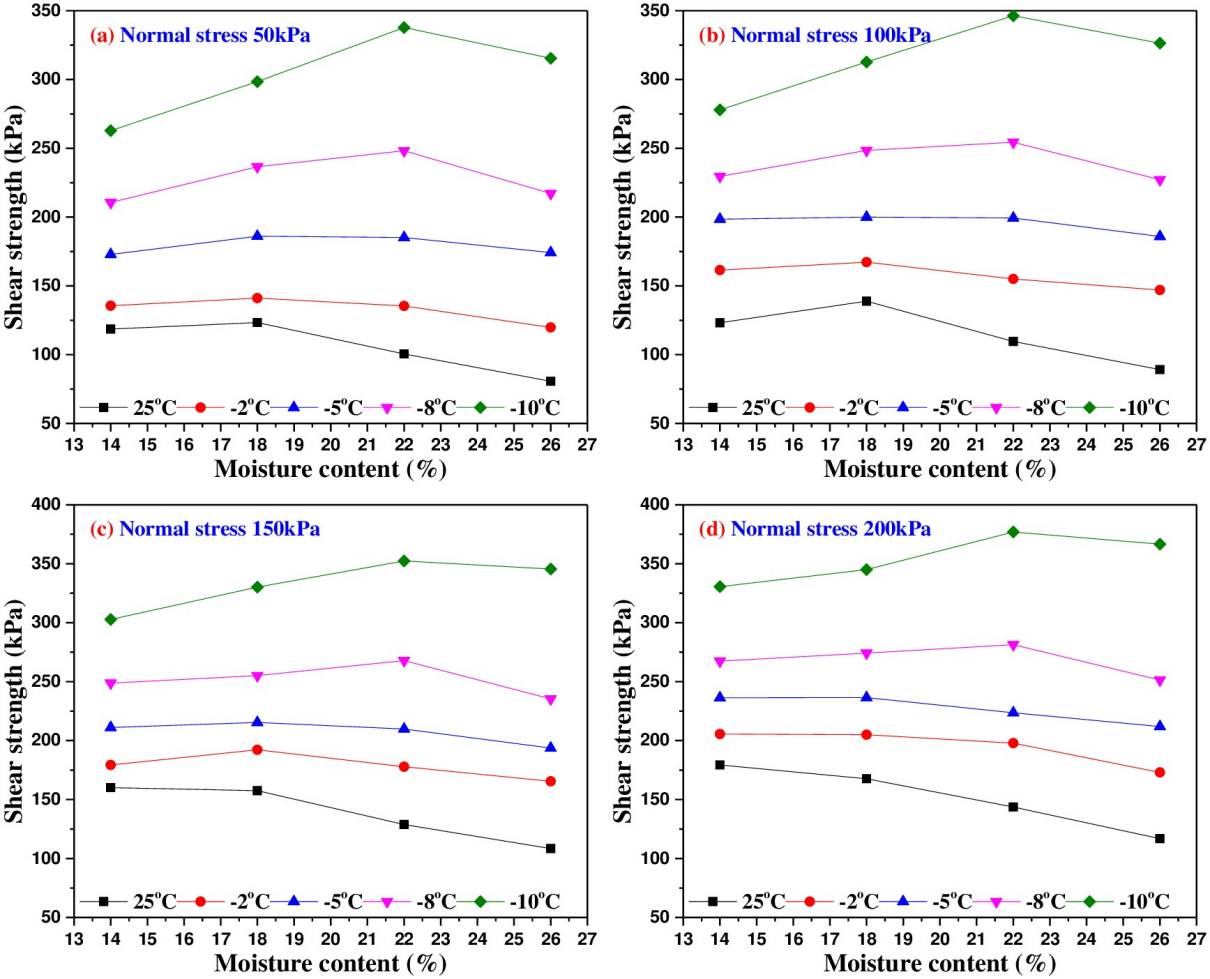
The relationship curves of shear strength and moisture content under different normal stresses.

It can be seen from Fig 13 that the shear strength of the interface presents a curve distribution with the change of moisture content, and there is a critical moisture content (also known as the ultimate moisture content). When the moisture content is smaller to the critical moisture content, the shear strength gradually increases with the increase of moisture content. However, when the moisture content exceeds the critical moisture content, the shear strength of the interface decreases with the increase of moisture content, and the critical moisture content changes with the influence of test temperature. This test result has similar characteristics to Arenson’s experiment on the relationship of freezing strength and moisture content [24], which shows that as the moisture content reaches to 80%~90% of saturated moisture content, ice crystals will fill the pores in the most possible way during freezing, but there is no frost heave, and the cementation of soil particles and the freezing of contact material surface shall be ensured to the greatest extent. When the moisture content is 26%, the moisture content exceeds the saturated water content, and the frost heave of ice occurs, which weakens the cementation of soil skeleton and the freezing effect of concrete surface. Therefore, when the moisture content of frozen soil is bigger than the critical moisture content, the shear strength of the interface decreases, and finally approaches the shear strength of the ice-concrete interface.

## 5. Analysis of shear strength parameters of interface

The direct shear test data of frozen soil-concrete interface show that the shear strength is affected by normal stress (σ), freezing temperature (*T*) and moisture content (*θ*). It can be seen from Fig 11 that the shear strength of the interface increases approximately linearly with the increase of normal stress. According to Mohr-Coulomb criterion, the shear strength (*τ*) of frozen soil-concrete interface can also be expressed as,

τ=σtanφ+c (1)

Where, the shear strength parameters cohesion (*c*) and internal friction angle (*φ*) are the functions of freezing temperature and moisture content. If the relationship of shear strength parameters, moisture content and freezing temperature is found out, the strength criterion of frozen soil-concrete interface can be obtained.

### 5.1 Effect of freezing temperature on shear strength parameters

**Fig 14:**
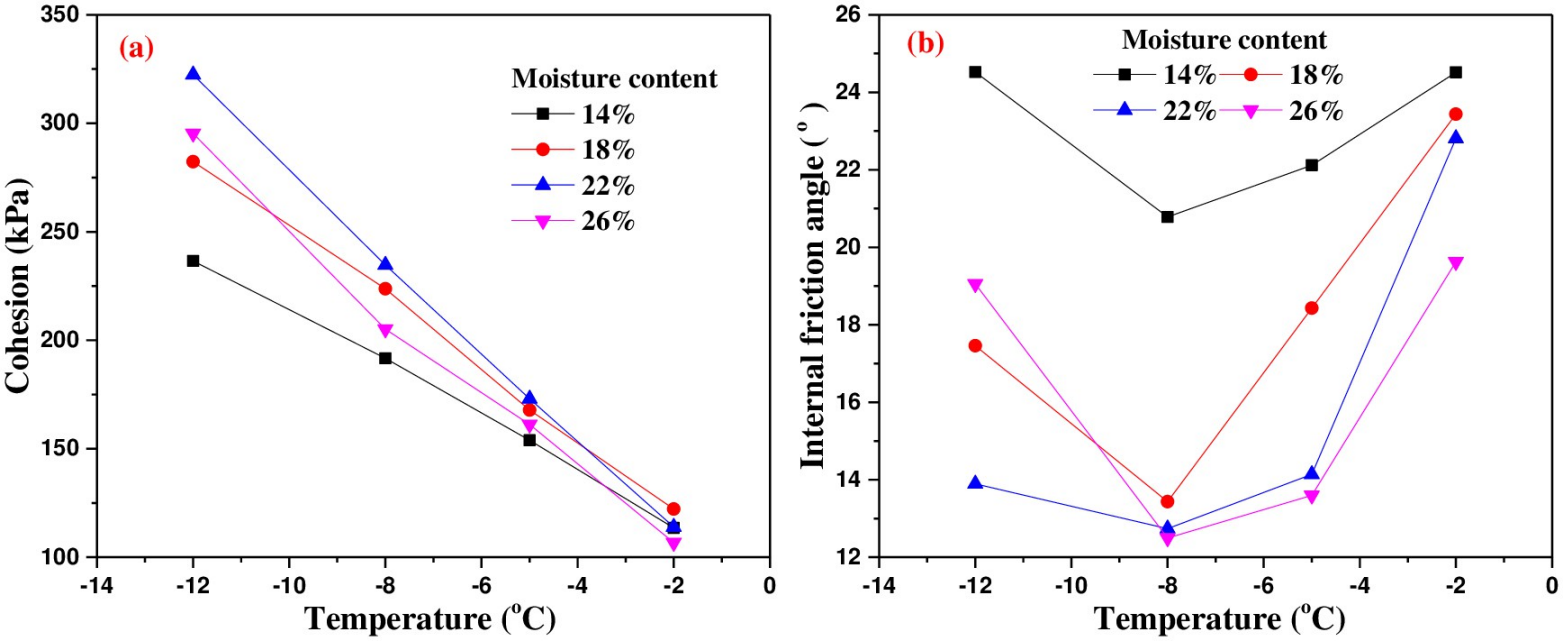
Variation of shear strength parameters with freezing temperature.

By substituting the shear strength of the interface under various test conditions into Eq (1), the relationship between cohesion and internal friction angle with temperature can be obtained as shown in Fig 14. With the decrease of freezing temperature, the cohesion of interface increases gradually. The existing studies have shown that the cohesion of unsaturated frozen soil consists of three parts: the initial cohesion of thawed soil, capillary cohesion and ice cementation [25]. The capillary cohesion is that the unfrozen water content decreases and the matrix suction in soil increases, which results in the enhancement of capillary cohesion. Ice cementation is the main component of the cohesion of unsaturated frozen soil and saturated frozen soil. On the one hand, with the decrease of freezing temperature, the unfrozen water content decreases and the ice crystal content increases. On the other hand, the ice crystal strength increases with the decrease of temperature. Therefore, the decrease of temperature can increase the cohesion of the interface, and the cohesion increases approximately linearly with the decrease of freezing temperature.

The internal friction angle of the frozen soil-concrete interface consists of three parts: the surface friction between soil particles and concrete, the surface friction of soil particles, and the bite force generated by the embedding and interlocking among particles. With the decrease of freezing temperature, the unfrozen water content gradually decreases and the unfrozen water film becomes thinner. During the water ice phase transition, the force on the soil particle skeleton is increased by the volume expansion of porous ice. The embedding and interlocking among soil particles are reduced by the damage of porous ice, and the reduction of internal friction angle is significant. The bigger the moisture content, the more obvious the frost heave effect of ice, the smaller the internal friction angle is. With the decrease of freezing temperature, the internal friction angle first decreases and then increases, which changes in a parabola.

### 5.2 Effect of moisture content on shear strength parameters

**Fig 15:**
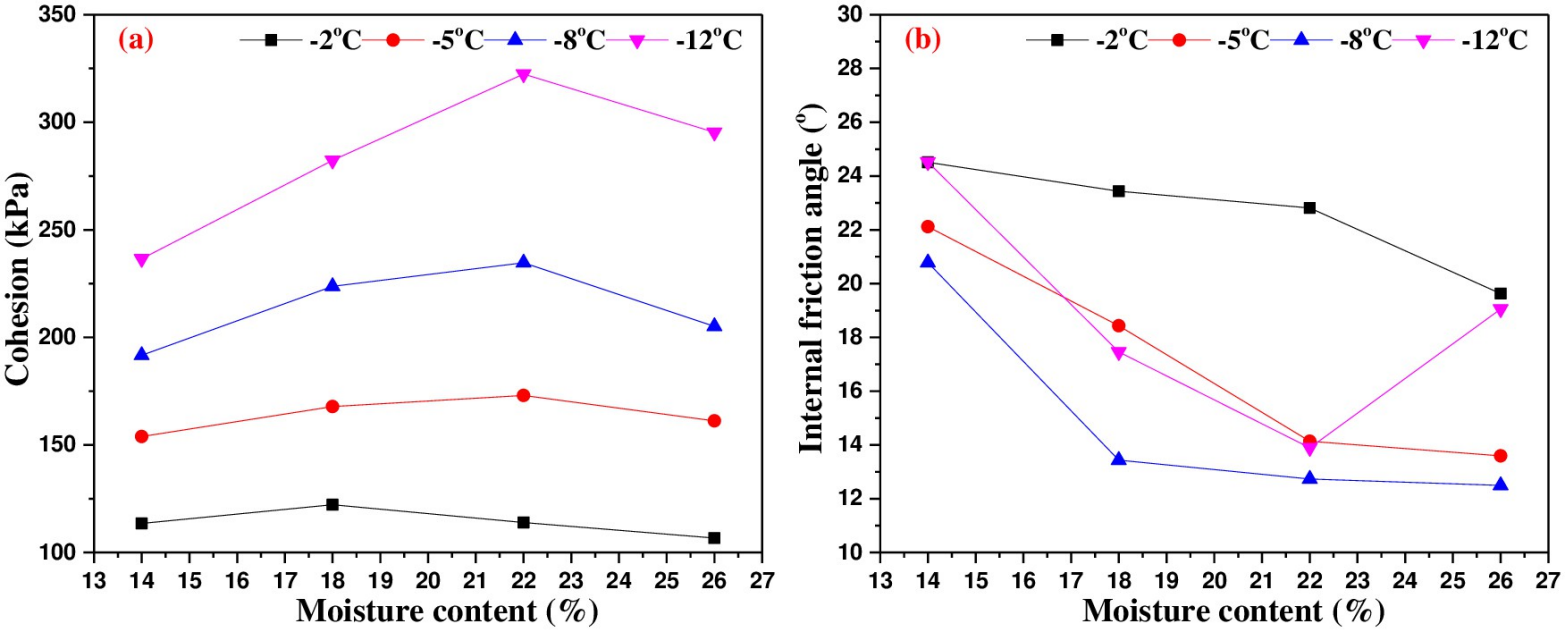
Variation curves of shear strength parameters with moisture content.

Fig 15 is the variation curves of cohesion and internal friction angle of interface with moisture content. It can be seen from Fig 15a that the cohesion of the interface increases and then decreases with the increase of moisture content. When the moisture content is close to the saturated state of soil, the contact area between concrete and ice in soil reaches the largest, the degree of cementation reaches the best, so the cohesion reaches the maximum. The cohesion presents a parabolic distribution with the increase of moisture content. And it can be seen from Fig 15b, the internal friction angle decreases with the increase of moisture content. With the moisture content increasing, ice crystals and unfrozen water gradually fill the pores near the contact surface, which results in the reduction of the contact area between soil particles and concrete, and the resulting friction is reduced accordingly. At freezing temperature, both minerals and water migrate in saturated core samples, which results in real-time changes in the freezing temperature of saturated soil samples[26]. At -8°C, this migration reaches stability the fastest and the unfrozen water content also decreases the fastest. This leads to a decrease in the embedding and interlocking effects between soil particles due to the damage of porous ice, and the decrease in internal friction angle is also the most significant. In addition, the filling effect of unfrozen water and ice crystal reduces the interface roughness and the bite force caused by the mutual embedding and interlocking between soil particles and concrete surface particles, which also leads to the decrease of internal friction angle with the increase of moisture content. The internal friction angle is approximately exponential with the increase of moisture content.

### 5.3 Multivariate regression of strength parameters

According to the analysis of shear strength parameters of interface, the cohesion and internal friction angle are functions of temperature and moisture content, which can be expressed as:

$$c=f_{1}(T,\theta )$$ (2)

$$\varphi =f_{2}(T,\theta )$$ (3)

Based on the experimental data, the specific function form can be fitted by multiple regression method. Multiple regression is a statistical method to reflect the change law between a dependent variable and multiple independent variables. Taking the simple linear regression as an example, it is assumed that *k* groups of experiments are carried out. The independent variables *x*_1_, *x*_2_,…, *x*_*p*_ have *k* groups of data, and the dependent variable *y* also has *k* numerical values. The relationship between independent variable and dependent variable is described by linear relationship:

$$y_{i}=a_{0}+a_{1}x_{1i}+a_{2}x_{2i}+\cdots +a_{p}x_{pi}\;(i=1,2,\cdots ,k)$$ (4)

Where, *a*_1_, *a*_2_,…, *a*_*p*_ are coefficients to be solved. The coefficients are calculated by the least square method to make the fitting function as close as possible to the actual test data.

The regression analysis of shear strength parameters is based on the relationship between cohesion, internal friction angle, temperature and moisture content. With the decrease of freezing temperature, the cohesion changes linearly and the internal friction angle changes parabola. Both cohesion and internal friction angle have a parabolic relationship with moisture content. Therefore, the internal friction angle and cohesion can be expressed as,

$$c=a_{0}+a_{1}T+a_{2}\theta^{2}+a_{3}\theta$$ (5)

$$\varphi =b_{0}+b_{1}T+b_{2}T^{2}+b_{3}\theta +b_{4}\theta^{2}$$ (6)

The cohesion and internal friction angle are calculated by the least square method to make the fitting function as close as possible to the actual test data, and the fitting values of cohesion and internal friction angle in different temperature and moisture are shown in Tables 1 and 2, which shown that the fitting values are agreed with the measured values, the relative errors are less than 10%. The fitting parameters are shown in Tables 3 and 4.

**Table 1:** Multi factor fitting of cohesion.

Temperature *T* (°C)	Moisture content *θ* (%)	Cohesion *c*(kPa)	Fitting value of cohesion (kPa)	Relative error (%)
-2	14	113.49	103.24	-7.48
-5	14	153.88	142.50	-7.39
-8	14	191.65	193.19	0.81
-12	14	236.5	253.01	6.98
-2	18	122.21	121.95	-0.21
-5	18	167.86	171.21	1.99
-8	18	223.74	221.90	-0.82
-12	18	282.31	291.72	3.33
-2	22	113.96	118.62	3.55
-5	22	173.03	177.87	2.80
-8	22	234.71	228.57	-2.62
-12	22	322.43	298.39	-7.46
-2	26	106.73	113.23	6.09
-5	26	161.22	162.49	0.79
-8	26	205.09	213.18	3.95
-12	26	295.32	283.01	-4.17

**Table 2:** Multi factor fitting of internal friction angle.

Temperature *T* (°C)	Moisture content *θ* (%)	Internal friction angle *φ* (°)	Fitting value of internal friction angle (°)	Relative error (%)
-2	14	24.51	25.34	3.38
-5	14	22.12	21.55	-2.58
-8	14	20.78	19.70	-5.20
-12	14	24.52	23.36	-4.73
-2	18	23.44	22.53	-3.90
-5	18	18.43	16.74	-9.18
-8	18	13.43	14.89	10.85
-12	18	17.46	18.55	6.22
-2	22	22.81	20.26	-11.19
-5	22	14.14	14.47	2.33
-8	22	12.73	12.62	-0.87
-12	22	13.89	14.28	2.80
-2	26	19.63	20.54	4.61
-5	26	13.59	14.75	8.51
-8	26	12.49	12.89	3.26
-12	26	19.05	17.56	-7.84

**Table 3:** Multi factor fitting coefficient of cohesion.

*c* = *a*_0_ + *a*_1_*T* + *a*_2_*θ*^2^ + *a*_3_*θ* Related coefficient (R^2^): 0.9510				
Coefficient	*a*_0_	*a*_1_	*a*_2_	*a*_3_
Value	-212.8936	-15.8617	-0.6889	29.2236

**Table 4:** Multi factor fitting coefficient of internal friction coefficient.

*φ* = *b*_0_ + *b*_1_*T* + *b*_2_*T*^2^ + *b*_3_*θ* + *b*_4_*θ*^2^ Related coefficient (R^2^): 0.9211					
Coefficient	*b*_0_	*b*_1_	*b*_2_	*b*_3_	*b*_4_
Value	70.2716	3.4611	0.2188	-3.7481	0.0795

Substituting the fitting parameters in Tables 3 and 4 into Eqs (1), (5) and (6), the shear strength (*τ*_*p*_) criteria of frozen soil-concrete interface can be obtained as,

$$\left\{\begin{array}{l} \tau_{p}=\sigma \text{tan}\varphi +c\\ c=-212.8936-15.8617T+29.2236\theta -0.6889\theta^{2}\\ \varphi =70.2716+3.4611T+0.2188T^{2}-3.7481\theta +0.0795\theta^{2} \end{array}\right.$$ (7)

### 5.4 Direct shear constitutive model of frozen soil-concrete interface

According to the shear stress and shear displacement curve, the relationship of shear stress and shear displacement can be divided into elastic deformation stage, plastic deformation stage and sliding failure stage. In order to establish the whole process expression of shear stress and shear displacement relationship, the hyperbolic model is used to approximately describe the elastic-plastic development stage, and the shear strength is used to represent the failure stage, so the hyperbolic function of shear stress and shear displacement is expressed as,

$$\tau =\frac{s}{a+bs}$$ (8)

When the shear stress reaches the shear strength (*τ* = *τ*_*p*_), then the shear stress and shear displacement is expressed as,

$$s=\frac{a\tau_{p}}{1-b\tau_{p}}$$ (9)

Where, *a* and *b* are fitting parameters, *τ* is shear stress, *s* is shear displacement.

Then for shear stress and shear displacement of frozen soil-concrete interface, the relationship model can be described as,

$$\tau =\left\{\begin{array}{ll} \frac{s}{a+bs} & \left(s\leq \frac{a\tau_{p}}{1-b\tau_{p}}\right)\\ \tau_{p} & \left(s>\frac{a\tau_{p}}{1-b\tau_{p}}\right) \end{array}\right.$$ (10)

**Fig 16:**
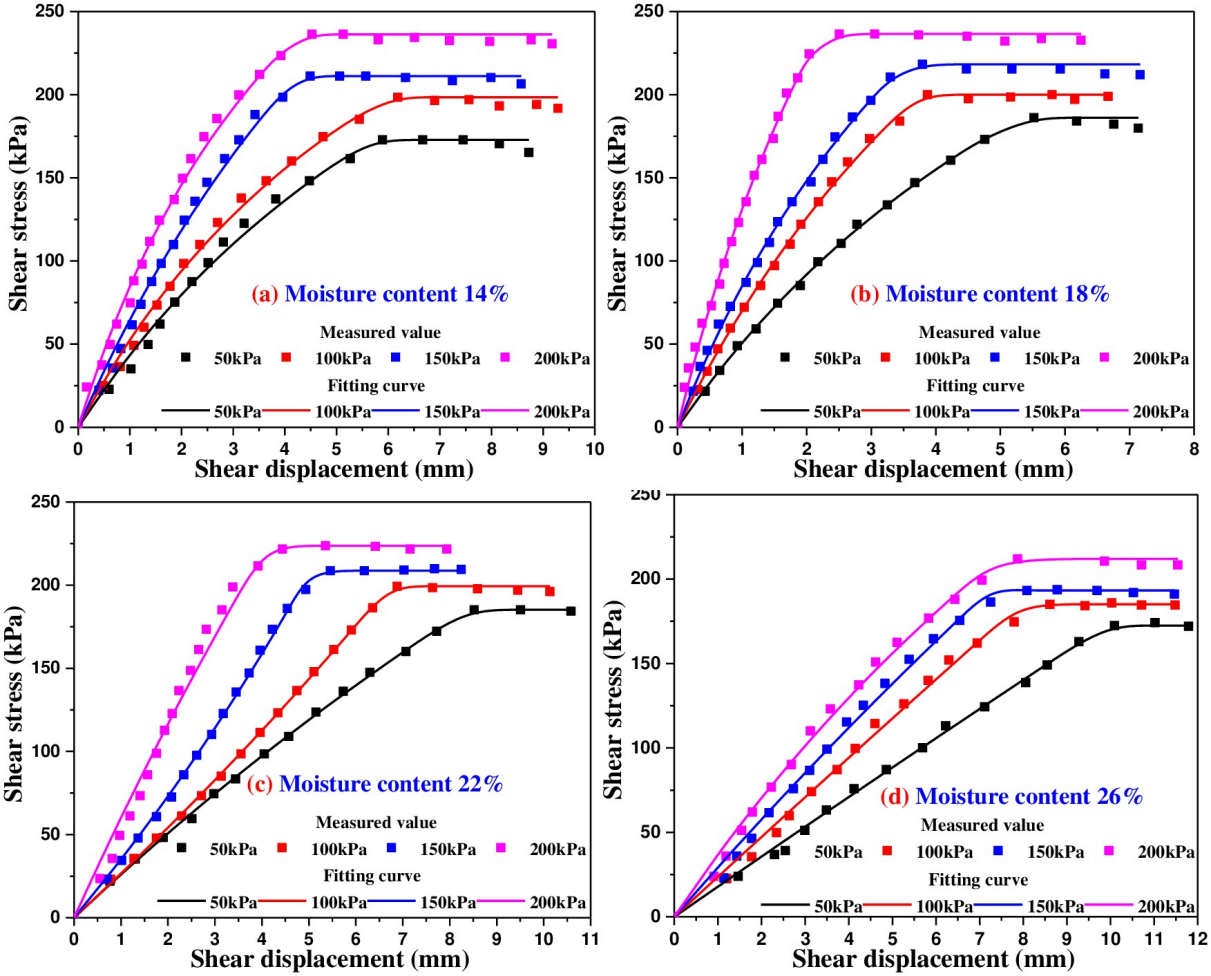
Comparison between calculated result and measured result of shear stress and shear displacement at -5°C.

The regression analysis of hyperbolic parameter is carried out for the shear stress and shear displacement curve measured in the direct shear test. The stress displacement are calculated by using the subsection function of Eq (10), and the curves of shear stress and shear displacement can be obtained as shown in Fig 16 (Taking the shear stress and shear displacement curve of soil-concrete interface at -5°C as an example). It can be concluded that the piecewise combination of hyperbolic function and linear function can be better consistent with the shear stress and shear displacement of frozen soil-concrete interface.

### 5.5 Direct shear failure mechanism of frozen loess-concrete interface

**Fig 17:**
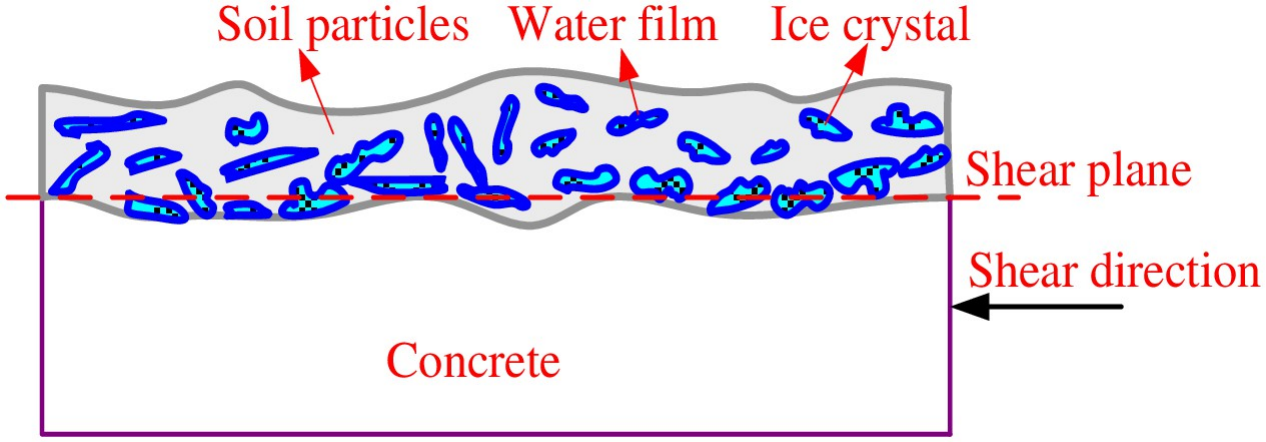
Direct shear mechanism of frozen soil-concrete interface.

The frozen soil-concrete interface is affected by the interaction of soil particles, concrete surface and ice crystal in the shear test process. It is considered that the shear strength of frozen soil-concrete interface is affected by the strength of ice, ice cementation, the surface friction of soil particles, the cohesion of soil particles, the friction of soil-concrete surface, the bite force generated by the embedding and interlocking between particles, etc. The shear mechanism of the interface is shown in Fig 17. Ice cementation is the most important factor for controlling the strength of frozen soil. However, ice cementation is restricted by ice content (the greater the ice content, the more cemented soil particles), the action of unfrozen water film of ice-soil particles and ice-concrete surface. According to the formation source of pore ice, pore ice is mainly formed by freezing free water, capillary water and some adsorbed water in soil.

With the increase of soil moisture content, the pore ice content increases after freezing. The more ice cemented soil-concrete particles, the greater the shear strength of the interface. When the moisture content of soil sample exceeds the saturated moisture content, the pore water mainly exists in the form of unfrozen water film (adsorption water film at room temperature). With the increase of moisture content, the unfrozen water film thickens, and the pore ice cementation weakens for soil particles and concrete particles. This is because the thickness of unfrozen water film determines the cementation strength of ice-soil particles and ice-concrete particles. And the thickness of unfrozen water film is approximately linear with freezing temperature. The lower the freezing temperature, the thinner the unfrozen water film [27]. The ice strength is mainly affected by freezing temperature, and with the decrease of freezing temperature, the fluidity of hydrogen atoms in ice crystal lattice will decrease, which results in the increase of ice strength [28].

The interface friction includes the surface friction of soil-concrete particles, the surface friction of soil particles and the bite force caused by the embedding and interlocking among particles. The friction among soil particles, the surface friction between soil-concrete particles is affected by the normal stress. The greater the normal stress is, the more obvious the friction effect is. The bite force generated by the embedding and interlocking between particles is obviously affected by the initial moisture content. When the initial moisture content is smaller, the soil sample freezes into pore ice, the volume expansion of water-ice phase transition has little effect on the soil skeleton, and the friction force on the interface is larger. As moisture content exceeds the saturated moisture content, more water is frozen into ice resulting in larger volume expansion, which affects the bite force formed by the embedding and interlocking among particles, and reduces the friction force of the interface.

## 6. Conclusion

Loess and high-strength plain concrete are made into soil-concrete samples with different moisture content, then the direct shear test of frozen soil-concrete interface is carried out under different freezing temperature, normal stress and moisture content by using a large pile-soil shear experimental device in cryogenic Laboratory. the influence law of different factors on the shear strength of interface is revealed, the stress displacement constitutive equation is proposed, and the shear strength criterion is established with considering the effects of freezing temperature and moisture content on cohesion and internal friction angle. The following conclusions can be obtained as,

1. The curve shape of shear stress and shear displacement of the interface in the direct shear test shows obvious segmentation, it divided into elastic deformation stage, plastic deformation stage and sliding failure stage successively.
2. The shear strength of frozen soil-concrete interface is affected by normal stress, freezing temperature and moisture content. The shear strength increases linearly with the increase of normal pressure, the shear strength increases linearly with the decrease of freezing temperature, and the shear strength increases and then decreases with the increase of moisture content.
3. The shear strength parameters of the interface are affected by freezing temperature and moisture content. With the decrease of freezing temperature, the cohesion increases linearly, while the internal friction angle decreases and then increases. With the increase of moisture content, the cohesion first increases and then decreases, while the internal friction angle decreases and then increases. The shear strength criterion is obtained by multiple regression method.
4. The shear stress and shear displacement relationship of frozen soil-concrete interface can be well simulated by using the piecewise combination of hyperbolic function and linear function.
5. The direct shear failure mechanism of frozen soil-concrete interface can be attributed to the strength of ice, the ice cementation, the cohesion of soil particles, the surface friction of soil-concrete particles, the surface friction among soil particles, the biting force caused by the embedding and interlocking among particles, etc.

## Supporting information

S1 Table Formula symbol summary table. (DOCX)

S1 Dataset Graph data. (XLS)

## References

[1] Riska K. Mechanics of Geomaterial Interfaces. 1995.

[2] Ga Zhang, Jianmin Zhang. State of the art: Mechanical behavior of soil–structure interface. Progress in Natural Science, 2009, 19: 1187–1196. doi: 10.1016/j.pnsc.2008.09.012

[3] Aldaeef Abdulghader A., Rayhani Mohammad T. Interface shear strength characteristics of steel piles in frozen clay under varying exposure temperature. Soils and Foundations, 2019, 59(6): 2110–2124. doi: 10.1016/j.sandf.2019.11.003

[4] Suguang Xiao, Suleiman Muhannad T., Al-Khawajac Mohammed. Investigation of effects of temperature cycles on soil-concrete interface behavior using direct shear tests. Soils and Foundations, 2019, 59(5): 1213–1227. doi: 10.1016/j.sandf.2019.04.009

[5] Ng C W W, Shi C., Gunawan A., Laloui L. Centrifuge modelling of energy piles subjected to heating and cooling cycles in clay. Géotechnique Letters, 2014, 4: 310–316. doi: 10.1680/geolett.14.00063

[6] Yavari Neda, Tangm Anh Minh, Pereira Jean-Michel, Hassen Ghazi. Experimental study on the mechanical behaviour of a heat exchanger pile using physical modelling. Acta Geotechnica, 2014, 9(3): 385–398. doi: 10.1007/s11440-014-0310-7

[7] Taha A, Fall M. Shear behavior of sensitive marine clay-concrete interfaces. Journal of Geotechnical Geoenvironmental Engineering, 2013, 139(4): 644–650. doi: 10.1061/(asce)gt.1943-5606.0000795

[8] Feligha Marwa, Hammoud Farid, Belachia Mouloud, Nouaouria Mohamed Salah. Experimental investigation of frictional behavior between cohesive soils and solid materials using direct shear apparatus. Geotechnical and Geological Engineering, 2016, 34(2): 567–578. doi: 10.1007/s10706-015-9966-5

[9] Su Li-Jun, Zhou Wan-Huan, Chen Wei-Bin, Jie Xixi. Effects of relative roughness and mean particle size on the shear strength of sand-steel interface, Measurement, 2018, 122: 339–346.

[10] Xuan Wang, Hao Cheng, Peng Yan, Jiasheng Zhang. The influence of roughness on cyclic and post-cyclic shear behavior of red clay-concrete interface subjected to up to 1000 cycles. Construction and Building Materials, 2021, 273: 121718. doi: 10.1016/j.conbuildmat.2020.121718

[11] Kou Hailei, Diao Wenzhou, Zhang Wangchun, Zheng Jingbin, Ni Pengpeng, JANG Bo-An, et al. Experimental study of interface shearing between calcareous sand and steel plate considering surface roughness and particle size. Applied Ocean Research, 2021, 107: 102490. doi: 10.1016/j.apor.2020.102490

[12] Lee Joonyong, Kim Youngseok, Chio Changho. A study for adfreeze bond strength developed between weathered granite soils and aluminum plate[J]. Journal of the Korean Geo-Environmental Society, 2013, 14(12): 23–30. doi: 10.14481/jkges.2013.14.12.023

[13] Yavari Neda, Tang Anh Minh, Pereira Jean-Michel, Hassen Ghazi. Effect of temperature on the shear strength of soils and soil-structure interface. Canadian Geotechnical Journal, 2016, 53(7): 1186–1194. doi: 10.1139/cgj-2015-0355

[14] Li Chunhong, Kong Gangqiang, Liu Hanlong, Abuel-Naga Hossam. Effect of Temperature on Behaviour of Red Clay/Structure Interface. Canadian Geotechnical Journal, 2018, 56(1): 126–134. doi: 10.1139/cgj-2017-0310

[15] Di Donna A, Ferrari A, Laloui L. Experimental investigations of the soil-concrete interface: physical mechanisms, cyclic mobilisation and behaviour at different temperatures. Canadian Geotechnical Journal, 2016, 53(4): 659–672. doi: 10.1139/cgj-2015-0294

[16] Yazdani Saeed, Helwany Sam, Olgun Guney. Influence of temperature on soil–pile interface shear strength. Geomechanics for Energy and the Environment, 2019, 18: 69–78. doi: 10.1016/j.gete.2018.08.001

[17] Lianzhen Zhao, Ping Yang, Laichang Zhang, Wang J.G. Cyclic direct shear behavior of an artificial frozen soil-structure interface under constant normal stress and sub-zero temperature. Cold Regions Science and Technology, 2017, 133: 70–81. doi: 10.1016/j.coldregions.2016.10.011

[18] Quanbin Shi, Ping Yang, Guoliang Wang. Experimental research on adfreezing strengthsat the interface between frozen fine sand and structures. Scientia Iranica, 2017, 25(2): 663–674. doi: 10.24200/sci.2017.20005

[19] Sheng Shi, Feng Zhang, Decheng Feng, Xiangtian Xu. Experimental investigation on shear characteristics of ice–frozen clay interface. Cold Regions Science and Technology, 2020, 176: 103090. doi: 10.1016/j.coldregions.2020.103090

[20] Jiankun Liu, Peng Lv, Yinghui Cui, Jingyu Liu. Experimental study on direct shear behavior of frozen soil–concrete interface. Cold Regions Science and Technology, 2014, 104–105: 1–6.

[21] Tianliang Wang, Haihang Wang, Tianfei Hu, Hong-Fng Song. Experimental study on the mechanical properties of soil-structure interface under frozen conditions using an improved roughness algorithm. Cold Regions Science and Technology, 2019, 158: 62–68.

[22] Pengfei He, Yanhu Mu, Zhaohui Yang, Wei Ma, Jianhua Dong, Yongting Huang. Freeze-thaw cycling impact on the shear behavior of frozen soil-concrete interface. Cold Regions Science and Technology, 2020, 173: 103024. doi: 10.1016/j.coldregions.2020.103024

[23] Pengfei He, Yanhu Mu, Wei Ma, YongTing Huang, JianHua Dong. Testing and modeling of frozen clayeconcrete interface behavior based on large-scale shear tests. Advances in Climate Change Research, 2021, 12: 83e94.

[24] Arenson Lukas U., Springman Sarah M., Sego David. The rheology of frozen soils. Applied Rheology, 2007, 17(1): 12147–12141.

[25] Zhenya Liu, Jiankun Liu, Xu Li, Jianhong Fang. Effect of capillary cohesion and ice cementation on strength and deformation of unsaturated frozen silty clay. Chinese Journal of Rock Mechanics and Engineering, 2018, 37(6): 1551–1559. doi: 10.13722/j.cnki.jrme.2017.1411

[26] Zhemin You, Wei Wen, Yuanming Lai, Mingyi Zhang, Jing Zhang. Model tests of the barrier measures on moisture and salt migration in soils subjected to freeze-thaw cycles. Cold Regions Science and Technology, 2022, 201: 103607. doi: 10.1016/j.coldregions.2022.103607

[27] Changgen Yan, Ting Wang, Hailiang Jia, Wei Xu, Fan Zi, Yue Tao, et al. Influence of the unfrozen water content on the shear strength of unsaturated silt during freezing and thawing. Chinese Journal of Rock Mechanics and Engineering, 2019, 38(6): 1252–1260.

[28] Xiaozu Xu, Jiacheng Wang, Lixin Zhang. Frozen Soil Physics[M]. Beijing: Science Press, 2010.

