# Supplementary material for: Nonlinear shear characteristics of frozen loess-concrete interface
Source: PLoS One. 2023 Aug 15;18(8):e0290025. doi: 10.1371/journal.pone.0290025 (PMC10426950; doi:10.1371/journal.pone.0290025)
Supplement: S1 Table — (DOCX) [file pone.0290025.s001.docx]

Table. 5 Formula symbol summary table

| Symbols | Implication |
| --- | --- |
| *c* | cohesion |
| σ | Normal stress |
| τ | shear stress |
| $\phi$ | internal friction angle |
| *a*_1_, *a*_2_,..., *a_p_* | coefficient |
| *T* | temperature |
| *θ* | moisture content |
| *τ_p_* | shear strength |
| *a,b* | fitting parameters |
| *s* | shear displacement |
